# The Pentachlorophenol Metabolite Tetrachlorohydroquinone Induces Massive ROS and Prolonged p-ERK Expression in Splenocytes, Leading to Inhibition of Apoptosis and Necrotic Cell Death

**DOI:** 10.1371/journal.pone.0089483

**Published:** 2014-02-26

**Authors:** Hsiu-Min Chen, Ben-Zhan Zhu, Rong-Jane Chen, Bour-Jr. Wang, Ying-Jan Wang

**Affiliations:** 1 Department of Environmental and Occupational Health, National Cheng Kung University, Medical College, Tainan, Taiwan; 2 State Key Laboratory of Environmental Chemistry and Ecotoxicology, Research Center for Eco-Environmental Sciences, Chinese Academy of Sciences, Beijing, China; 3 Department of Occupational and Environmental Medicine, National Cheng Kung University Hospital, Tainan, Taiwan; 4 Department of Cosmetic Science and Institute of Cosmetic Science, Chia Nan University of Pharmacy and Science, Tainan, Taiwan; Taipei Medical University, Taiwan

## Abstract

Pentachlorophenol (PCP) has been used extensively as a biocide and a wood preservative and has been reported to be immunosuppressive in rodents and humans. Tetrachlorohydroquinone (TCHQ) is a major metabolite of PCP. TCHQ has been identified as the main cause of PCP-induced genotoxicity due to reactive oxidant stress (ROS). However, the precise mechanisms associated with the immunotoxic effects of PCP and TCHQ remain unclear. The aim of this study was to examine the effects of PCP and TCHQ on the induction of ROS and injury to primary mouse splenocytes. Our results shown that TCHQ was more toxic than PCP and that a high dose of TCHQ led to necrotic cell death of the splenocytes through induction of massive and sudden ROS and prolonged ROS-triggered ERK activation. Inhibition of ROS production by *N*-acetyl-cysteine (NAC) partially restored the mitochondrial membrane potential, inhibited ERK activity, elevated caspase-3 activity and PARP cleavage, and, eventually, switched the TCHQ-induced necrosis to apoptosis. We suggest that prolonged ERK activation is essential for TCHQ-induced necrosis, and that ROS play a pivotal role in the different TCHQ-induced cell death mechanisms.

## Introduction

Pentachlorophenol (PCP) and its salt are used extensively as biocides and wood preservatives [1]–[3]. The annual production of PCP has been estimated to be approximately 46 million pounds in the United States [4]. Due to improper disposal, PCP has become an environmental pollutant and is now considered to be ubiquitous [5]. PCP is not prone to degradation because of its stable aromatic ring system and high chlorine content [6]. It has been indicated that PCP is persistent with a half-life of up to 200 days in water systems [7]. In addition, the half-life of PCP ranges from 33 hours to 16 days in humans [8]. In rodents, chronic exposure is associated with adverse effects on a variety of biological systems, including the immune system [9]–[11]. In humans, malignant lymphoma and leukemia have been associated with occupational exposure to PCP [12]. Tetrachlorohydroquinone (TCHQ) is a major toxic metabolite of PCP. In the presence of oxygen, TCHQ can lead to production of superoxide radicals through a cycle of autoxidation and reduction between TCHQ and its corresponding semiquinone radical under certain physiological conditions [13]. Furthermore, the dismutation of the superoxide radicals to H_2_O_2_ can lead to hydroxyl radical formation in the presence of ferrous or cupreous ions [14], [15]. Thus, reactive oxygen species (ROS) are believed to be involved in the toxic effects of TCHQ.

Exposure to chemical agents can result in cell damage and death. The survival or death of the exposed cells is often determined by proliferative status, DNA repair enzyme capacity, and the ability to induce proteins that either promote or inhibit the cell death processes [16]. Mammalian cells demonstrate a high degree of flexibility in cell death responses, as is reflected by a variety of mechanisms, including apoptosis, autophagy and necrosis [17]–[19]. The precise nature of the molecular events associated with the various cell death pathways is not well understood. In general, apoptosis is an active process of cell destruction with specific defining morphologic and molecular features that leads to orderly cell disassembly. Necrosis, in contrast, is thought to be the passive consequence of massive cell damage and hence accidental, uncontrolled, and harmful by nature. However, recent studies suggest that this simplistic view of necrosis may need to be redefined [20], [21].

The mitogen-activated protein kinase (MAPK) cascades are activated by various cellular stresses and growth factors and are involved in various biological responses such as differentiation, proliferation and cell death [22], [23]. In mammals, MAPK cascades are composed of three distinct signaling modules: JNK, p38 MAPK and extracellular signal-regulated kinase (ERK). Upon cytokine or growth factor stimulation, MAPK activation is usually rapid and transient. However, genotoxic stresses such as UV or γ-irradiation induce long-lasting or prolonged MAPK activation. Transient MAPK activation has been reported to be associated with gene expression, proliferation or differentiation, whereas prolonged MAPK activation promotes cell death in a cell type- and stimulus-dependent manner [24]–[26]. In various pathological conditions such as ischemia, accumulation of excessive ROS induces apoptosis or necrosis by activating MAPK, caspase cascades, and/or disruption of mitochondrial membrane potential [27]–[29]. However, it remains unclear whether ROS play a critical role in environmental toxicant-induced prolonged MAPK activation and its role in triggering apoptosis or necrosis.

Our previous studies show that TCHQ can induce DNA strand breakage in mammalian cells, glutathione conjugate formation, and the depletion of glutathione content in liver tissue [30], [31]. In addition, with PCP and TCHQ, oxidative stress and different cell death mechanisms can be induced in rats and human hepatoma or bladder cell lines [31], [32]. Both PCP and TCHQ were shown to be tumor promoters in our previous study using a mouse skin model in CD-1 mice [33]. The generation of ROS and alterations in Bcl-2 gene expression could be related to the possible mechanisms of TCHQ-induced tumor promotion [34]. The administration of an antioxidant prevented ROS generation, cytotoxicity, genotoxicity and apoptotic cell death in cells treated with TCHQ. Furthermore, activation of MAPK may be involved in TCHQ-mediated apoptosis [35]. Recently, we also found that PCP is immunotoxic in vivo. Exposure of mice to PCP led to mild changes in cytokine secretion and suppression of immunoglobulins IgG and IgM [36]. The observations of immunosuppressive effects in animals, as well as in humans exposed to PCP-containing wood preservatives have led to the suspicion that PCP exerts a damaging influence on the immune system [9], [37]–[39]. Nevertheless, the precise underlying mechanisms related to the immunotoxic effects of PCP and TCHQ have not yet been clarified.

*N*-acetyl-cysteine (NAC) has been widely used as an antioxidant in vivo and in vitro. The ability of NAC to protect cells against oxidant damage could be due to both of its ability to maintain intracellular glutathione (GSH) concentrations and scavenge oxidants. NAC can be readily deacetylated in cells to yield L-cysteine, which is a precursor of GSH synthesis [40]. In addition, because of its sulfhydryl group, NAC is an excellent scavenger of hydroxyl radical (·OH) and a powerful scavenger of hypochlorous acid (HOCl). It also reacts slowly with hydrogen peroxide (H_2_O_2_), but it is a fairly poor scavenger of superoxide (O_2_^−^) [41]. It has been shown that systemic administration of NAC prevents brain GSH depletion in animal studies [42]–[44], which benefits in a variety of neurodegenerative disease models [45]–[47]. NAC decreases oxidative stress and rescues cells from apoptosis [48], [49]. Also, NAC has been shown to prevent radiation-induced DNA breaks [40].

In the present study, we examined the effects of PCP and TCHQ on the induction of ROS and injury to primary murine splenocytes. The cytotoxic doses and cell death mechanisms including apoptosis and necrosis in the splenocytes were determined and characterized both by morphologic and biochemical markers. We also examined the protective effect of NAC against the PCP/TCHQ-induced oxidative damage and consequent cell death. How cell injury could trigger the program of events that results in apoptosis or necrosis is not well understood. To evaluate the mechanisms for these effects, we further examined the activation of ERK, one of the MAPK cascades, during the processes of apoptosis or necrosis induced by PCP or TCHQ.

## Materials and Methods

### Chemicals

PCP (purity >98%) and TCHQ (purity approx. 98%) were purchased from Sigma Chemical Co. (St. Louis, MO, USA). The PCP used in the present study was the original compound and not the sodium salt form. Both of them were prepared in DMSO. RPMI-1640 medium, 3-(4,5-Dimethylthiazol-2-yl)-2,5-diphenyltetrazolium bromide (MTT) and phenol-chloroform-isoamyl alcohol (25∶24∶1, v/v/v) were purchased from Sigma-Aldrich (St. Louis, MO, USA). Antibiotic was purchased from Gibco BRL (Grand Island, NY, USA). Fetal calf serum was purchased from HyClone (South Logan, UT, USA). Annexin V-FITC was purchased from Becton Dickinson (San Jose, CA, USA). DCFH-DA and DiOC_6_ were purchased from Molecular Probes (Eugene, OR, USA). Antibodies against ERK1/2, phospho-ERK1/2 (Thr202/Tyr204), poly(ADP-ribose)polymerase (PARP), ERK1/2 inhibitor U0126, horseradish peroxidase-conjugated anti-mouse and anti-rabbit secondary antibodies were purchased from Cell Signaling (Beverly, MA, USA). Cleaved Caspase-3 antibodies were purchased from BioVision (Mountain View, CA, USA). Anti-β-actin antibody was purchased from Sigma Chemical Co.

### Ethics Statement

All animal experiments were conducted in strict accordance with the guidelines of our institute (the Guide for Care and Use of Laboratory Animals, National Cheng Kung University Medical College). The animal handling procedures were approved by the Institutional Animal Care and Use Committee of National Cheng Kung University (Approval No: 98112). All surgery was performed under anesthesia, and all efforts were made to minimize suffering.

### Isolation of Splenocytes

Male ICR mice were purchased from the animal center of the National Cheng Kung University Medical College, and kept under quarantine in that animal center for 1 week before starting the experiments. The mice were housed four per cage at 24±2°C and 50±10% relative humidity, subjected to a 12-h light/12-h dark cycle and fed with a Purina chow and water ad libitum. Eight to twelve-week-old mice were sacrificed and the spleens were immediately removed and single cell suspensions were prepared. Specifically, the spleens were placed in RPMI-1640 medium supplemented with antibiotics (100 U/mL of penicillin and 100 µg/mL of streptomycin) and 10% heat-inactivated fetal calf serum (referred to as complete medium). The spleens were gently minced and passed through fine wire mesh to remove all debris. The contaminating RBCs were removed by treating with lysis buffer (0.16 M NH_4_Cl and 0.17 M Tris buffer mixed in the ratio 9∶1, pH 7.2) for 5 min at room temperature. The cells were centrifuged at 1500 rpm for 5 min and washed with PBS. The resuspended cells were collected and maintained in complete medium. The cells were incubated in a humidified atmosphere containing 5% CO_2_ at 37°C. The cell viability of the freshly isolated cells was greater than 98% in each experiment, as determined by trypan blue exclusion.

### MTT Assay

The cell viability was determined using the MTT assay. The freshly prepared splenocytes were seeded at a density of 2×10^5^ cells/well into 96-well culture plates containing various concentrations of PCP or TCHQ, which were dissolved in DMSO. DMSO alone replaced the toxins in the untreated control wells. After the indicated time points, 100 µL of MTT solution (5 mg/mL in PBS) was added to each well and incubated for 2 hr. The MTT solution was then removed and 100 µl of DMSO was added to dissolve the dark blue crystals thoroughly. The absorbance was measured at 570 nm using an Emax precision microplate reader (Molecular Devices Instruments, USA).

### DNA Fragmentation Assay

DNA fragmentation was assessed by gel electrophoresis. The freshly prepared splenocytes were cultured at a density of 2×10^6^ cells in 10-cm dishes and treated with PCP or TCHQ for 2 hr. The cells were harvested and washed twice with ice-cold PBS. The cell pellets were lysed in a buffer containing 50 mM Tris-HCl (pH 8.0), 10 mM EDTA, 0.5% sarkocyl (N-laurylsarkosin) and protease K (0.2 mg/mL) at 50°C for 16 hr. A solution of 5 µL RNase A (10 mg/mL) was added and the incubation was continued for 1 hr longer at 50°C. The DNA was extracted with phenol-chloroform-isoamyl alcohol (25∶24∶1, v/v/v), loading buffer was added, and the samples were electrophoresed in a 2% agarose gel. The gel was stained with ethidium bromide and visualized under UV light in an imaging instrument (Alphalmager™ 2200 System, Alpha Innotech Corporation, San Leandro, CA, USA).

### Flow Cytometry

#### 1. Cell cycle analysis

The control and treated splenocytes were collected, washed with ice-cold PBS and fixed with 75% ethanol at 4°C for 16 hr. After fixation, cells were washed twice with ice-cold PBS and incubated in 1 mL of a solution containing 0.1% Triton X-100, RNase A (39.5 µg/mL) and PI (20 µg/mL) for 30 min. The fluorescence emitted from the PI-DNA complex was measured at 488 nm excitation/600 nm emission using a FACScan instrument (Becton Dickinson, San Jose, CA, USA), and the percentage of cells below the G1 peak (subG0/G1 fraction) was analyzed using the WinMDI software (Becton Dickson, San Jose, CA, USA).

#### 2. Cell death detection

To analyze PCP- and TCHQ-induced cell death, Annexin V/PI staining was performed and used as an indicator of apoptosis and necrosis. The control and treated splenocytes were collected, washed with ice-cold PBS and centrifuged at 2000 rpm for 5 min. The cells were resuspended in 1 mL of 1×Annexin V-binding buffer (10 mM HEPES (pH7.4), 0.14 M NaCl and 2.5 mM CaCl_2_) and 100 µL of the suspension was transferred to a Falcon tube. Next, 5 µL of Annexin V-FITC and 2 µL of PI (1 mg/mL) were added and mixed thoroughly followed by a 15-min incubation. After the 15 min incubation at room temperature, 1×Annexin V-binding buffer (400 µL) was added to stop the reaction. The fluorescence was measured using the FACScan and analyzed using the WinMDI software.

#### 3. ROS production measurement

Intracellular reactive oxygen species (ROS) production was monitored by flow cytometry. After treatment with PCP or TCHQ, the cells were loaded with DCFH-DA at a final concentration of 5 µM and incubated for 30 min at 37°C. The harvested cells were washed and resuspended in ice-cold PBS. The fluorescence was analyzed using the FACScan and analyzed using the WinMDI software.

#### 4. Mitochondria membrane potential analysis

The change in mitochondrial transmembrane potential was monitored by flow cytometry. The PCP- or TCHQ-treated cells were incubated with DiOC_6_ at a final concentration of 30 nM for 30 min at 37°C. The cells were collected and then washed and resuspended in ice-cold PBS. The fluorescence was measured using the FACScan and analyzed using the WinMDI software.

### Western Blot

PCP- and TCHQ-treated and untreated splenocytes were collected and washed with ice-cold PBS. Total cellular protein lysates were prepared by lysing cells with freshly prepared extraction buffer supplemented with protease and phosphatase inhibitors (20 mM Tris-HCl (pH7.9), 140 mM NaCl, 10% glycerol, 10 µg/mL aprotinin, 10 µg/mL leupeptin, 5 µg/mL pepstatin A, 2 mM PMSF and 5 mM DTT for at least 20 min on ice. The lysates were centrifuged at 15,000 rpm for 30 min at 4°C, and the extracts were harvested for further analysis. The cytoplasmic and nuclear fractions were prepared by suspending the cells in ice-cold hypotonic buffer (10 mM NaH_2_PO_4_ (pH7.8), 10 mM NaF, 5 mM EDTA, 5 mM MgCl_2_, 1 mM PMSF and 1% NP-40) for 30 min on ice. The cells were centrifuged at 4000×g for 15 min at 4°C. The supernatants containing the cytoplasmic fractions were collected. The pellets were resuspended in hypotonic buffer supplemented with 1% NaCl and kept on ice for 30 min with periodic vortexing. The lysates were centrifuged at 14,000×g for 30 min at 4°C, and the supernatants containing the nuclear proteins were collected. The protein concentrations were determined using the Bio-Rad protein assay (Bio-Rad Laboratories). The samples were then boiled for 5 min and subjected to SDS-PAGE on 8∼12% (w/v) gels. The proteins were transblotted onto Immobilon-P-PVDF membranes (Millipore) and detected using rabbit anti-activated caspase-3 polyclonal Ab, rabbit anti-PARP mAb, rabbit anti-p44/42 MAPK (ERK1/2) mAb, rabbit anti-phospho-p44/42 MAPK (ERK1/2) (Thr202/Tyr204) mAb or rabbit anti-β-actin mAb. The membranes were incubated with horseradish peroxidase-conjugated antibodies appropriate to the species of the primary antibody and the immunoreactive bands were visualized using the ECL chemiluminescent detection system (PerkinElmer Life Science, MA, USA) and BioMax LightFilm (Eastman Kodak, New Haven, CT, USA) according to the manufacturer’s instructions. The protein quantitation was performed using a computer densitometer (AlphaImager™ 2200 System, Alpha Innotech Corporation, San Leandro, CA, USA).

### Caspase Activity

Caspase-3 activity was quantified using a fluorogenic assay, CaspACETM Assay System (Promega, WI, USA), according to the manufacturer’s instructions. Briefly, 50 µg of total protein was incubated with 50 mM caspase-3-specific substrate, Ac-Asp-Glu-Val-Asp-AMC (Ac-DEVD-AMC), at 30°C for 1 hr. The cleavage of this substrate by caspase-3 released the free fluoro-chrome 7-amino-4-methyl coumarin (AMC) which was measured at 360 nm excitation/460 nm emission in a Fluoroskan Ascent FL microplate fluorometer (Thermo Scientific, USA).

### Electron Microscopy (EM)

The cells were fixed with a solution containing 2.5% glutaraldehyde and 2% paraformaldehyde (in 0.1 M cacodylate buffer, pH 7.3) for 1 hr. After fixation, the samples were post-fixed in the same buffer containing 1% OsO4 for 30 min. Ultrathin sections were then observed using a transmission electron microscope (JEOL JEM-1200EX, Japan) at 100 kV.

### Statistics

The experimental data are expressed as means ± SD. The statistical significance was determined by using the Student’s *t-test* for comparison between the means or one-way analysis of variance with *post-hoc Tukey HSD test* and the differences were considered significant when *p*<0.05. In addition, the *Kolmogorov-Smirnov (K-S) test* was used to confirm all of our raw data have a normal distribution and the non-parametric *Kruskal-wallis test* was used to confirm results in cases where data did not satisfy normality tests.

## Results

### Cytotoxicity and Different Forms of Cell Death Induced by TCHQ or PCP Treatment of Splenocytes

**Figure 1:**
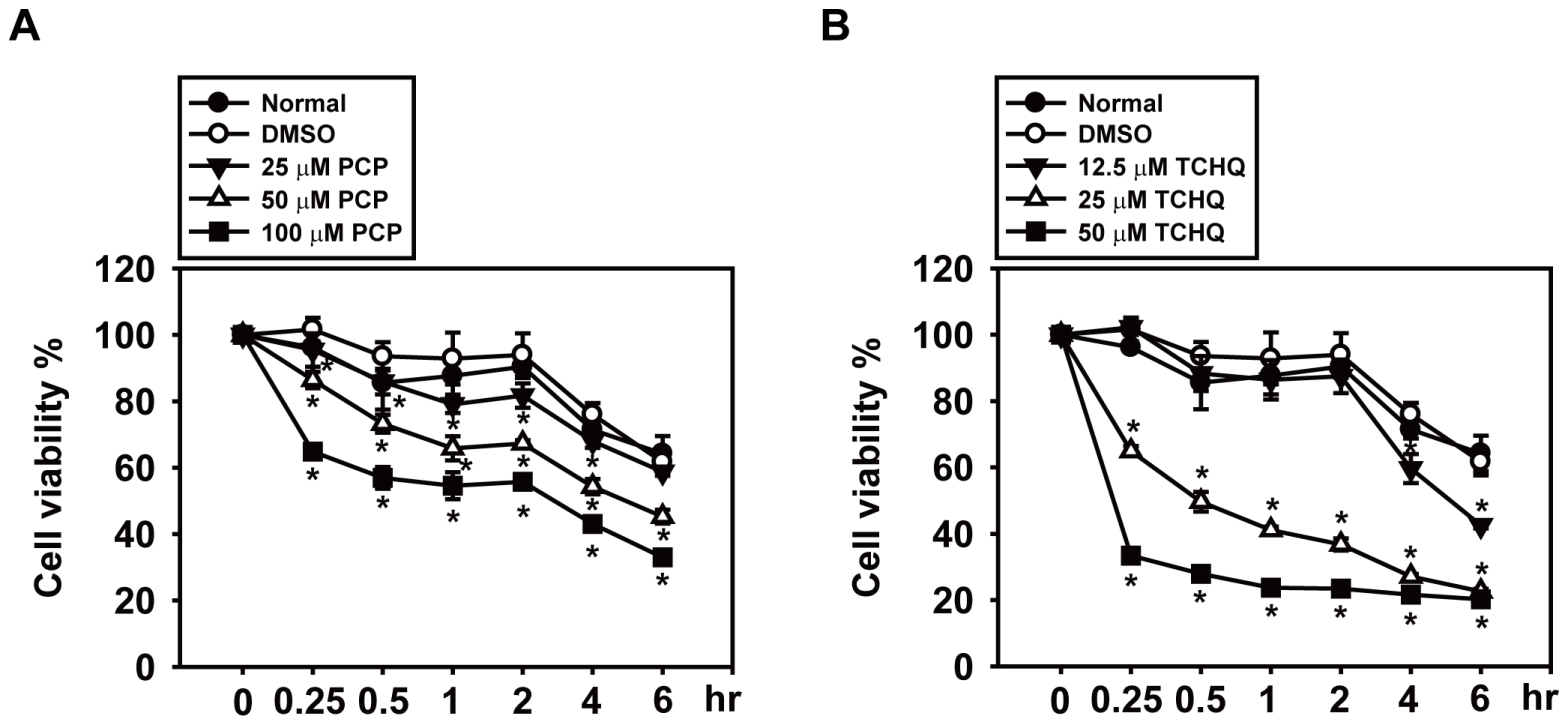
PCP and TCHQ inhibit cell viability in a dose- and time-dependent manner in splenocytes. Freshly isolated mouse splenocytes were treated with various concentrations of PCP or TCHQ dissolved in DMSO for the time periods indicated. The cell viability was measured using the MTT assay. Values are the mean ± SD from six replicates of three independent experiments. Statistical analysis was performed using the two-tailed Student’s *t*-test. *, p<0.05 versus DMSO control group at the time period indicated.

To exam the toxic effects of PCP and TCHQ on the splenocytes and their dose dependency, splenocytes isolated from male ICR mice were treated with different doses of PCP or TCHQ for 0.25–6 hr. The MTT assay revealed that both PCP and TCHQ induce obvious cytotoxicity in the splenocytes in a time- and dose-dependent manner (Fig. 1A and 1B). TCHQ was showed more toxic than PCP. For example, following treatment with 50 µM PCP or TCHQ for 30 min, the viability of the splenocytes was decreased to 73.25% and 27.94%, respectively. A longer treatment (6 hr) with same doses of PCP or TCHQ led to enhanced toxicity as indicated by the significantly decreased viability (45.24% and 20.24%, respectively) (Fig. 1).

**Figure 2:**
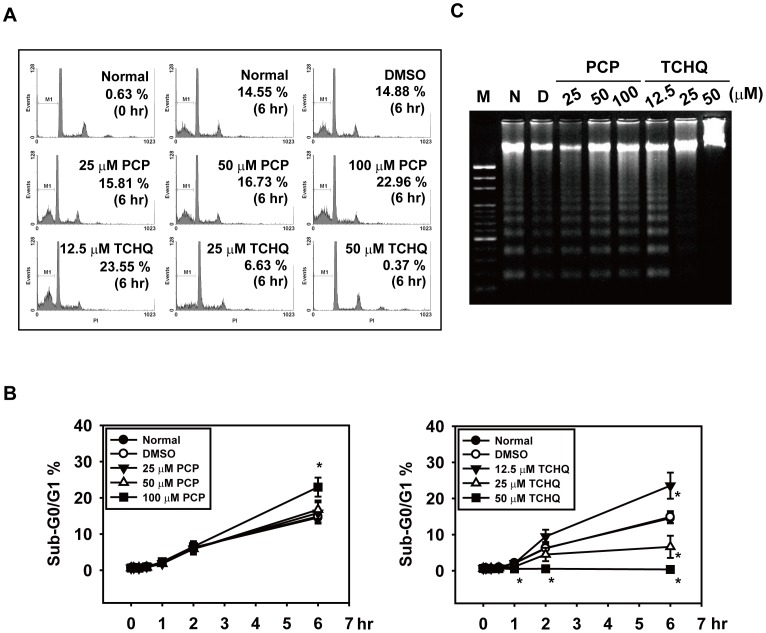
TCHQ treatment in higher doses inhibits sub-diploid DNA content and DNA ladder in splenocytes. (A, B) After treatment with PCP or TCHQ for 0, 5 min, 15 min, 30 min, 1 hr, 2 hr or 6 hr, the splenocytes were collected and stained with PI. The cell cycle distribution was analyzed using the FACScan (Becton Dickinson, San Jose, CA, USA) and analyzed using WinMDI software (Becton Dickson, San Jose, CA, USA). Cells with sub-diploid DNA content (Sub-G0/G1 phase) were defined as apoptotic. Values are the mean ± SD from at least three separate experiments. The statistical analysis was performed using the two-tailed Student’s *t*-test. *, p<0.05 versus DMSO control group at the time period indicated. (C) To further confirm these effects, analysis of DNA fragmentation in splenocytes was performed. Following PCP or TCHQ treatment for 2 hr, the cells were lysed and the DNA was extracted and electrophoresed on a 2% agarose gel. The gel was stained with EtBr and photographed. M: marker; N: normal splenocytes group; D: DMSO control group.

**Figure 3:**
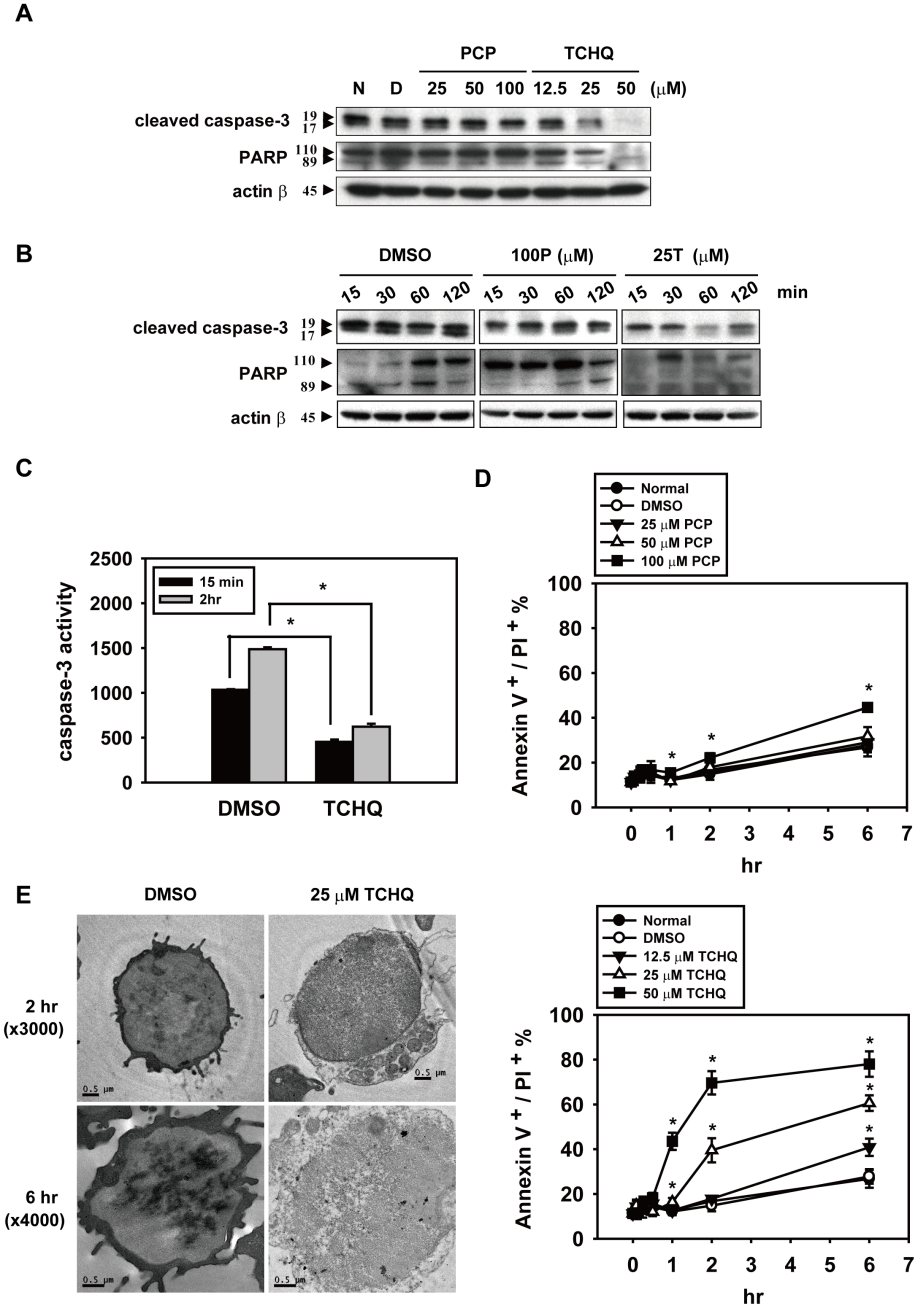
High dose treatment with TCHQ results in inhibition of apoptosis in splenocytes. (A) Freshly isolated mouse splenocytes were treated with indicated concentrations of PCP or TCHQ for 2 hr. Total protein lysates were analyzed for the expression of cleaved caspase-3 and PARP by western blot. β-actin was served as a loading control. N: normal group; D: DMSO control group. The expression of the target proteins at the indicated time points are shown in (B). Capase-3 activity was detected by a fluorogenic assay, CaspACETM Assay System (Promega, WI, USA), and Caspase-3 released free fluoro-chrome 7-amino-4-methyl coumarin (AMC) was measured at 360 nm excitation/460 nm emission in a Fluoroskan Ascent FL microplate fluorometer (Thermo Scientific, USA). Statistical analysis was performed using a two-tailed Student’s *t*-test. *, p<0.05. (D) Cell death analysis was performed using Annexin V and PI double-staining. The treated splenocytes were stained for detection of PS with Annexin V-FITC and for DNA content with PI. The necrotic cells were characterized by loss of plasma membrane integrity (annexin V**+/**PI**+**). The fluorescence was measured using a FACScan and analyzed by WinMDI software. The statistical analysis was performed using a two-tailed Student’s *t*-test. *, p<0.05 versus DMSO control group at the time period indicated. (E) Transmission electron microscopy (JEOL JEM-1200EX, Japan) was used to examine cell morphology. Scale bar: 0.5 µm.

We next examined the mechanism of the cell death induced by PCP and TCHQ in the splenocytes. Several cellular and biochemical markers were applied to characterize apoptosis, including the percentage of sub-G0/G1 phase, DNA laddering, cleavage of caspase-3, caspase-3 activity, and degradation of PARP, as shown in Fig. 2 and Fig. 3. Spontaneous apoptosis could be observed in untreated splenocytes. Treatment of the splenocytes with 100 µM PCP further enhanced the sub-G0/G1 percentage (from 14.88% to 22.96%) and the intensity of the DNA laddering (Fig. 2A and 2C). However, different responses were found in the splenocytes treated with TCHQ. Treatment with the lower dose (12.5 µM) of TCHQ also increased the percentage of the sub-G0/G1 cells and DNA laddering as found in PCP, whereas higher doses of TCHQ (25 µM and 50 µM) dramatically decreased the spontaneous apoptosis of the splenocytes in a dose- and time-dependent manner (Fig. 2). The results of the analyses of the cleaved caspase-3 and PARP degradation with western blots and the assessment of caspase-3 activity by a fluorogenic assay further confirmed the inhibition of apoptosis in the splenocytes treated with the higher doses of TCHQ (Fig. 3A, 3B and 3C), implying that a cell death type other than apoptosis could be induced by TCHQ.

### Treatment with Higher Doses of TCHQ Induces Necrosis, Inhibiting Apoptosis in Splenocytes

It has been reported that necrotic cells can be defined as both annexin V and PI positive and caspase-3 activity negative [50], [51]. We therefore examined the degree of necrotic cell death in splenocyes treated with PCP or TCHQ. As shown in Fig. 3D, TCHQ significantly increased the double positive (Annexin V ^+^/PI ^+^) staining of the splenocytes in a dose- and time-dependent manner, whereas PCP slightly increased the double positive staining at 100 µM (Fig. 3D, upper panel). The results strongly indicated that TCHQ induced necrosis in splenocytes. We then examined the cellular morphology by electron microscopy. The splenocytes treated with 25 µM TCHQ for 2 and 6 hr showed increases in translucent cytoplasm, swelling organelles, increased dilation of the nuclei, cell volume, and disruption of the plasma membrane (Fig. 3E). These results indicated that the high dose of TCHQ induced more necrotic cell death than apoptosis in the splenocytes.

### TCHQ Induces Massive Intracellular ROS Production in a Short Time and Inhibits Expression of Proteins Involved in Apoptosis

**Figure 4:**
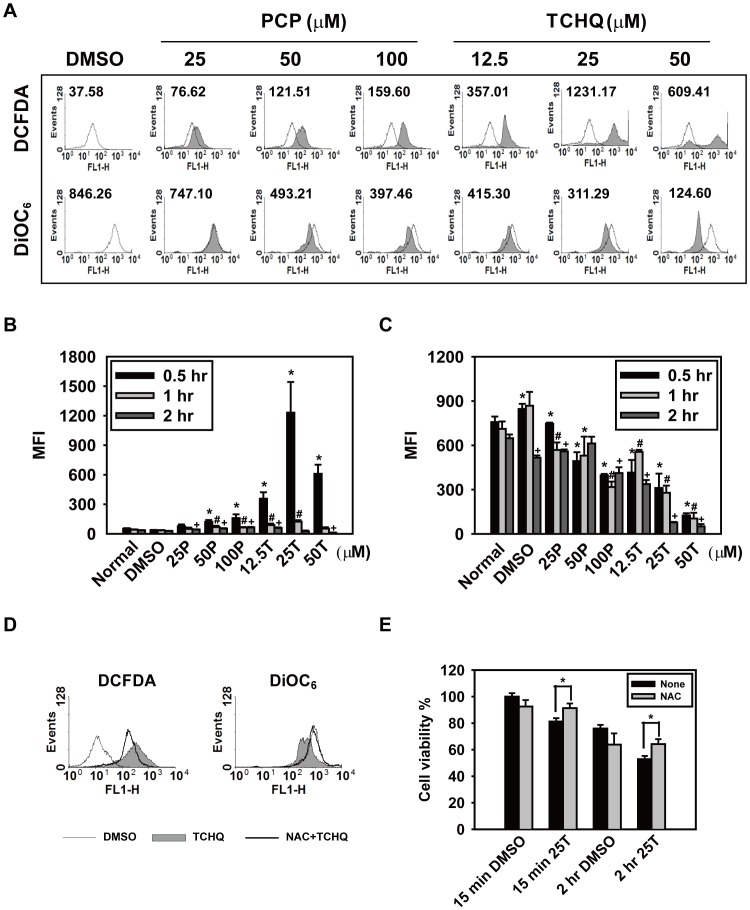
TCHQ induces massive intracellular ROS production and persistent attenuation of membrane potential in splenocytes. (A) Splenocytes were treated with a range of PCP or TCHQ for 2 hr. DCFDA (30 µM) was added to the cell suspensions for intracellular ROS detection and 30 nM DiOC_6_ was added for membrane potential analysis 30 min prior to the end of the exposures. After treatment, the cells were harvested and their fluorescence was analyzed using the FACScan and analyzed using WinMDI software. The data are shown as MFI. ROS production and the loss of membrane potential in splenocytes treated with PCP or TCHQ for the indicated periods of time were presented as (B) and (C), respectively. MFI: Mean Fluorescence Intensity. Statistical analysis was performed using the two-tailed Student’s *t*-test. *^, #, +^, p<0.05 versus DMSO control group in treatment at the time period indicated. (D) NAC was added as an antioxidant. Splenocytes with or without 5 mM NAC pretreatment for 1 hr were collected and washed with ice-cold PBS, followed by 25 µM TCHQ treatment for 30 min. ROS, membrane potential and cell death were measured as described above. Finally (E), cell viability was analyzed using the MTT assay. Statistical analysis was performed using the two-tailed Student’s *t*-test. *, p<0.05.

Previous studies indicated that TCHQ and its semiquinone counterpart can undergo redox cycling to induce the production of excessive ROS, which can result in damage to biological macromolecules such as DNA, leading to cytotoxicity and carcinogenicity [52]–[55]. Similarly, our previous report also indicated that TCHQ-induced cell death could be through a ROS-triggered signaling pathway [34], [35]. In some experimental scenarios, ROS production by mitochondrial respiratory complex I could lead to damage to mitochondria and result in the loss of mitochondrial transmembrane potential, which is crucial for necrosis [56]–[58]. Thus, we suggested that higher doses of TCHQ may induce necrotic cell death in splenocytes through ROS-triggered signaling. To test this hypothesis, splenocytes were treated with the indicated concentrations of PCP or TCHQ for 0.5, 1, and 2 hr, followed by the addition of 30 µM DCFDA, an fluorescence dye for detecting ROS. As shown in Fig. 4A and 4B, TCHQ, especially at the higher doses, significantly increased ROS production concurrent with a loss of mitochondria membrane potential was observed compared to the DMSO control and PCP-treated groups. We observed a strong increase in ROS production with a peak at 30 min in response to 25 µM TCHQ (Fig. 4B). A significant loss of mitochondrial membrane potential also occurred at 30 min with a persistent attenuation in splenocytes treated with higher doses of TCHQ for 0.5–2 hr, as detected by DiOC_6_ (Fig. 4C). These results implied that excessive ROS generation induced by TCHQ may play a pivotal role in the TCHQ-induced necrosis.

**Figure 5:**
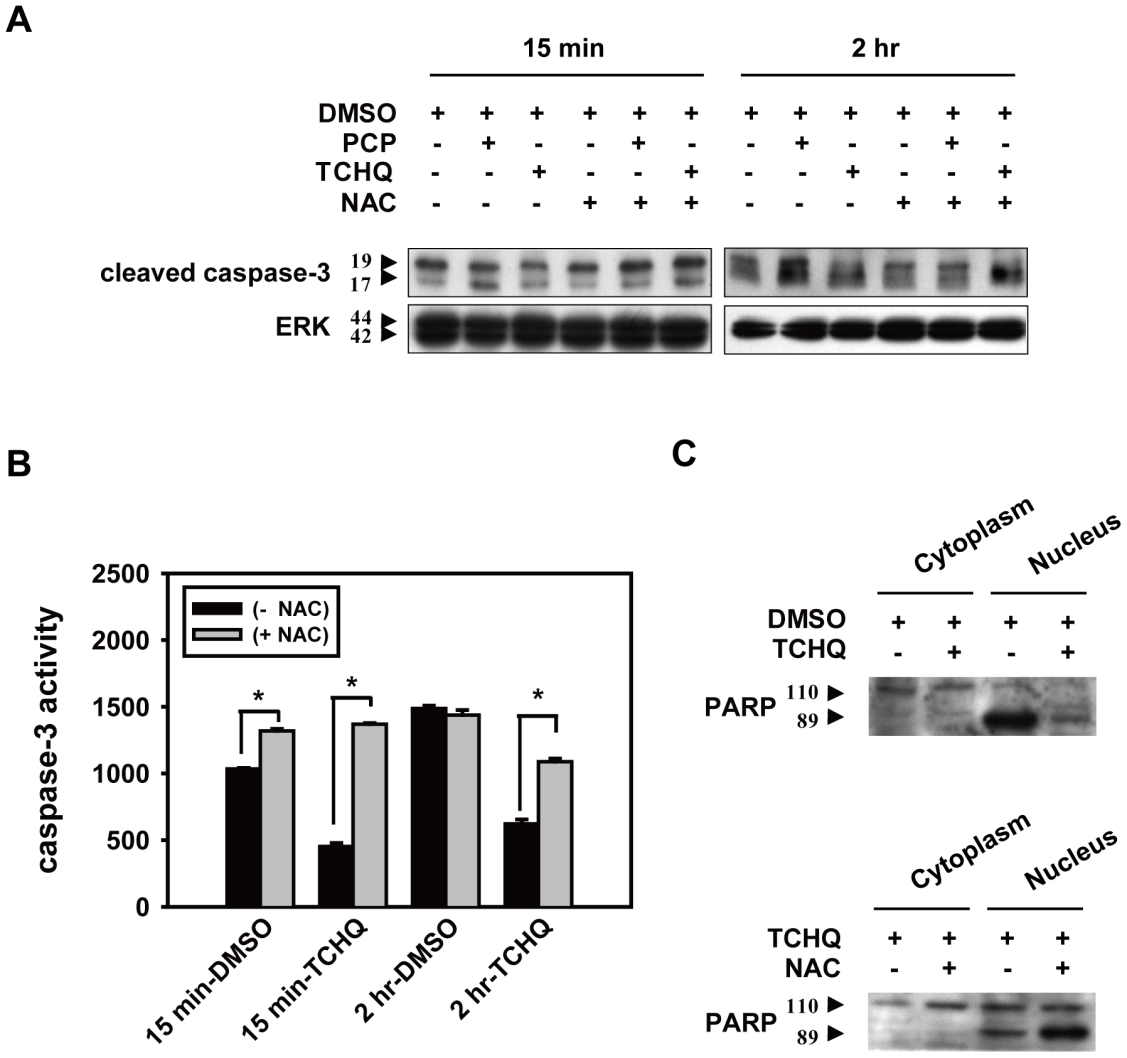
NAC prevents TCHQ-inhibited effects on cleaved caspase-3 and PARP and caspase-3 activity in splenocytes. (A) Splenocytes were pretreated with or without 5 mM NAC for 1 hr. After washing in ice-cold PBS, the cells were treated with 100 µM PCP or 25 µM TCHQ for 15 min or 2 hr, respectively. Total protein lysates were analyzed for the expression of cleaved caspase-3 by western blot. ERK expression served as a loading control. (C) Splenocytes pretreated for 1 hr with (lower panel) or without (upper panel) NAC, were treated with 25 µM TCHQ for 2 hr. Nuclear and cytosolic extracts were prepared and subjected to western blot analysis. (B) Capase-3 activity was detected using CaspACETM Assay System and was measured by Fluoroskan Ascent FL microplate fluorometer. Statistical analysis was performed using a two-tailed Student’s *t*-test. *, p<0.05.

NAC is one of the most common antioxidants used to investigate the effects of ROS in the pathogenesis of many oxidative stress-related diseases [59], [60]. Hence, splenocytes were pretreated with 5 mM NAC for 1 hr followed by treatment of 25 µM TCHQ to evaluate the importance of ROS. We found that pretreatment with NAC could lead to a significant reduction of ROS generation, prevented the changes in mitochondrial membrane potential (Fig. 4D), and partially prevented the loss of cell viability (Fig. 4E). These results implied that TCHQ-induced necrosis in splenocytes could be triggered by excessive ROS generation. Notably, blockage of ROS production by NAC attenuated the TCHQ-induced necrosis, but increased apoptosis as determined by expression of cleaved caspase-3 (Fig. 5A), caspase-3 activity (Fig. 5B), and degraded PARP expression compared to TCHQ alone (Fig. 5C). The results suggested that TCHQ-induced ROS may induce sudden stress to splenocytes and led to necrosis, thereby inhibiting apoptosis. NAC suppressed ROS generation and retarded TCHQ-induced necrosis, thus permitting the spontaneous apoptosis of the splenocytes.

### TCHQ Induces Prolonged p-ERK Expression that Might Mediate Apoptosis Inhibition

**Figure 6:**
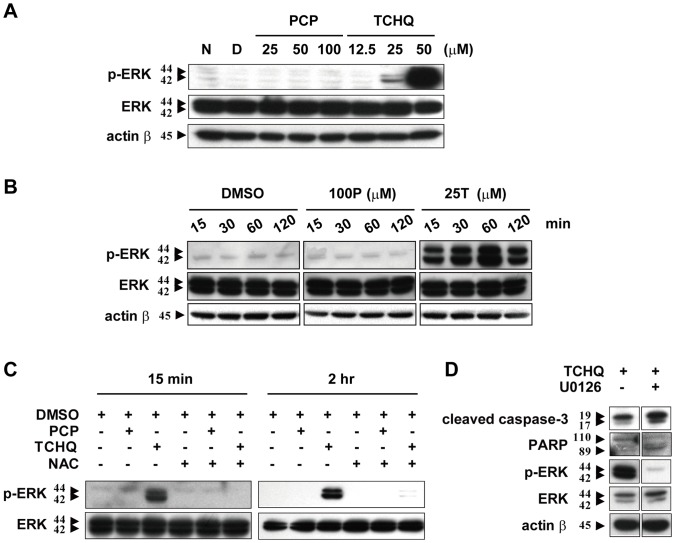
TCHQ-induced, ROS-mediated, prolonged p-ERK expression in splenocytes. (A) Freshly isolated splenocytes were treated with various concentrations of PCP or TCHQ for 2 hr. The total protein lysates were extracted to analyze the expression of p-ERK and ERK by western blot. β-actin was served as a loading control. N: normal group; D: DMSO control group. The protein expression for the indicated periods of time is presented as (B). (C) The splenocytes were pretreated with or without 5 mM NAC for 1 hr. After washing with ice-cold PBS, the cells were treated with 100 µM PCP or 25 µM TCHQ for 15 min or 2 hr. the Total protein lysates were extracted to analyze the expression of p-ERK and ERK by western blot. ERK served as a loading control. (D) The cells were pretreated with U0126 (10 µM) for 30 min followed by treatment of 25 µM TCHQ for 2 hr. Total cell lysates were prepared and immunoblotted using antibodies to cleaved caspase-3, PARP, p-ERK, ERK and β-actin. β-actin was served as a loading control. Statistical analysis was performed using a two-tailed Student’s *t*-test. *, p<0.05.

As reported previously, excessive accumulation of ROS induces apoptosis or necrosis by activating MAPK, caspase cascades, and/or disruption of mitochondria membrane potential [27], [61]. Thus, we tested whether TCHQ-induced ROS is associated with ERK activation. As shown in Fig. 6A and Fig. 6B, the higher doses of TCHQ caused a dose-dependent and prolonged ERK activation. However, PCP did not induce ERK activation, indicating the specific role of ERK in splenocytes after exposure to TCHQ. Pretreatment of the splenocytes with NAC significantly inhibited the ERK activation induced by 15 or 2 hr of TCHQ treatment (Fig. 6C). The results indicated that massive ROS production mediates the prolonged ERK activation in TCHQ-stimulated splenocyte. Blocking ERK activation with the selective MEK1/2 inhibitor U0126 resulted in increased expression of cleaved caspase-3 and degradation of PARP (Fig. 6D). These results indicated that TCHQ-induced massive ROS production resulting in prolonged ERK activity might be one of the signaling pathway leading to the inhibition of apoptosis.

**Figure 7:**
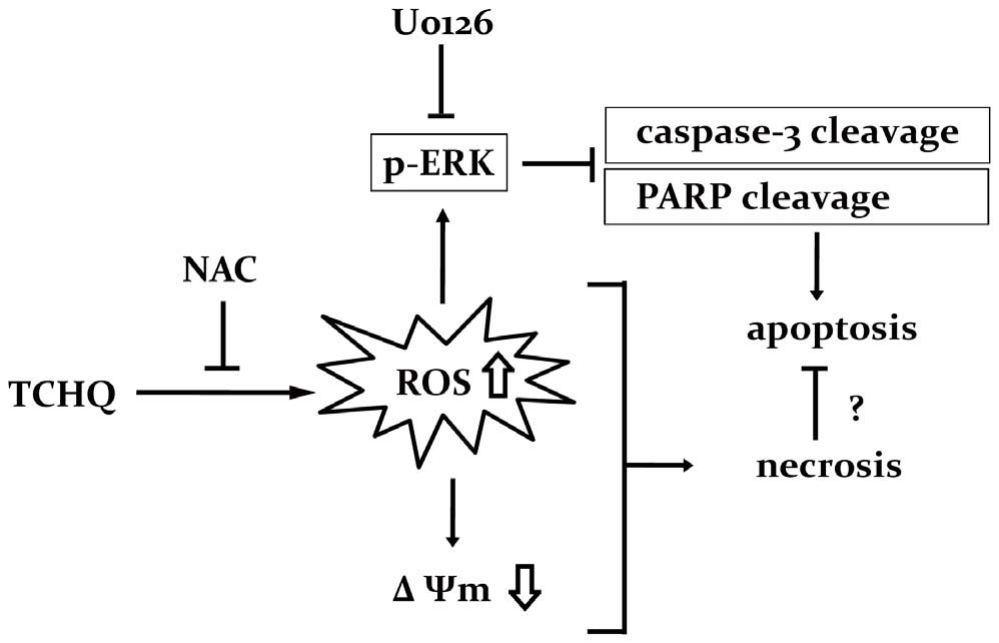
The probable cell death pathways mediated by high doses of TCHQ in splenocytes.

Our results indicate that high doses of TCHQ led to necrosis of splenocytes through induction of massive and sudden ROS and ROS-triggered prolonged ERK activation. The inhibition of ROS production by NAC can partially prevent the changes in mitochondrial membrane potential, inhibit ERK activity, elevate caspase-3 activity and PARP cleavage, and, eventually switch the TCHQ-induced cell death from necrosis to apoptosis. Similar to the effects of NAC, U0126 can also lead to the cleavage of caspase-3 and PARP by in habiting ERK activity. We suggested that prolonged ERK activation is essential for TCHQ-induced necrosis (Fig. 7).

## Discussion

The damage induced by PCP and TCHQ was investigated in freshly isolated splenocytes. The results suggest that PCP and lower doses of TCHQ induced apoptosis in splenocytes, but higher doses of TCHQ induced cell death more characteristic of necrosis than apoptosis. Apoptosis is a well-studied biochemical and morphological form of programmed cell death [18], [62], [63]. The rapid translocation and accumulation of the phospholipid phosphatidylserine (PS) on the outer leaflet of the plasma membrane is one of the early characteristics of apoptosis and is thought to trigger specific recognition and removal of these cells by phagocytes before lysis [64], [65]. Freshly isolated viable monocytes present only a few PS in the outer membrane leaflet, and the PS density is too low to induce phagocytosis [66]. Annexin V has a high affinity for PS and is used as an indicator of early apoptosis [67]. In our current study, some of the freshly isolated splenocytes already showed an annexin V^+^/PI^−^ phenotype (data not shown) with almost no appearance of sub-G0/G1 fraction, and the cell viability was greater than 98% determined by trypan blue exclusion (data not shown). The apoptotic characteristics could be observed within 2 hr after isolation. Thus, in this case, the freshly isolated splenocytes was viable, but spontaneously and progressively underwent apoptosis. However, apoptotic cells are not only cell that expose PS in the outer membrane; previous studies have also indicated that thymocytes and JB6 cells underwent necrosis show early exposure of PS at the outer leaflet of the continuous plasma membrane (annexin V^+^/PI^−^), which was suggested to be essential for phagocytic clearance [68]–[70]. Our current study indicated that exposure of splenocytes to 12.5 µM TCHQ resulted in apoptosis (sub-G0/G1 phase, DNA ladder, caspase-3 activity, PARP cleavage), whereas exposure to a higher level of TCHQ (25 µM) induced necrotic characteristics, as demonstrated by the significantly increased level of double positive (Annexin V ^+^/PI ^+^) staining and lacking of apoptotic characteristics (sub-G0/G1 phase, DNA ladder, caspase-3 activity, PARP cleavage). We propose that ROS generation in the exposed splenocytes could be involved, at least in part, in this phenomenon because ROS generation has been shown to be one of the toxic mechanisms induced by PCP and TCHQ [34], [35], [53], [71]. PCP- and TCHQ-induced ROS generation was observed in this current study. However, a sudden and massive ROS production was observed only when the cells were exposed to high levels of TCHQ (25 and 50 µM). Under these conditions, The ROS production was accompanied by a drastic decline in cell viability and the loss of mitochondrial membrane potential. Pretreatment with NAC can inhibit TCHQ-induced ROS production and also retard the loss of mitochondrial membrane potential. As a consequence, NAC was found to prevent the change in caspase-3 activity and the cleaved PARP expression in the splenocytes treated with TCHQ (Fig. 5).

An important area of future research is identification of the genes that are involved in the apoptotic program or other types of cell death. In fact, the findings that cell death occurs at a certain time and at certain locations during precise stages of normal development or metamorphosis imply that there are genes responsible for the occurrence of cell death [72]. In this study, it was found that the expression of the ERK gene increased and was significantly prolonged in cells treated with TCHQ. However, no significant changes were found in the cells treated with PCP. Consistent with these observations, Wispriyono et al. also reported that a significant level of ERK phosphorylation could be induced by TCHQ but not PCP in human Jurkat T cells [73]. The ERK pathway plays a major role in regulating cell growth and differentiation [74]–[77]. The activation of the ERK pathway is generally considered to be a survival signal induced by growth factors that reduce apoptotic signals [78], [79]. It has been reported that the ERK activation plays antiapoptotic roles: blocking apoptosis by inhibiting the activation of caspase-8, a key component of the death receptor pathway, which is induced by extrinsic stimulation of Fas, TNFR, and TRAIL receptor [80], [81]. Obviously, the increased phosphorylation of ERK in the splenocytes found in this study is not sufficient to protect them against the lethal metabolic alterations brought about by TCHQ. These results suggest that TCHQ-induced cell damage may induce a profound intracellular signal for the induction and activation of ERK, whereas the PCP-induced damage may not induce this signal. However, the basis for crosstalk between ERK activation and the apoptotic/necrotic cell death machinery remains largely unclear.

Another interesting result from this study is that a substantial part of splenocytes died by necrosis after higher doses of TCHQ stimulation, as assessed by electron microscopy and flow cytometry (Figure 3E). Furthermore, given that NAC only marginally affected the emergence of spontaneous apoptotic cells but substantially inhibited the high dose TCHQ-induced massive ROS generation and necrosis related changes (Figure 5), ROS could mainly, but not exclusively, contribute to necrotic cell death. ROS produced by mitochondria, or at the plasma membrane due to NADPH oxidases, are established pathophysiological regulators of gene transcription, proliferation, and apoptosis [82], [83]. Additionally, ROS production observed in response to a variety of chemical stimuli triggers necrosis via stimulation of lipid peroxidation and overall perturbation of the cellular redox status [84], [85]. Interestingly, low levels of oxidative stress favor apoptosis while excessive oxidative stress precludes caspase activation and drives cells toward necrosis [86], [87]. Induction of necrosis in lymphoid cells has been reported by Villena et al. showing that ceramide-induced necrotic cell death is linked to the loss of mitochondrial membrane potential, production of ROS, and intracellular ATP depletion [88]. In addition, it has been reported that accumulation of ROS could mediate prolonged MAPK activation and trigger necrotic cell death [61]. In our current study, we demonstrated the first time that massive ROS generation induced by an environmental toxicant triggered prolonged ERK activation and consequent necrosis in primary mouse splenocytes. Necrosis and apoptosis are distinct mechanisms of cell death with very different characteristics. A major difference between the two types of cell death is the involvement of the neighboring cells. The leakage of cellular contents by necrotic cells causes a pro-inflammatory response in the neighboring cells. In apoptotic cells, the internal and external membranes are preserved so that the cellular contents are safely sealed within the dying cells [89]. However, the distinction between apoptosis and necrosis in cell culture models can be confusing due to the lack of scavenging cells, and thus the phagocytic step after apoptosis may not occur. Therefore, it will be interesting to determine whether necrotic morphological changes are also observed in *in* *vivo* immune system exposed to TCHQ.

Occupational and household exposures to PCP have been associated with immune alterations in humans [37], [38]. In addition, long-term low-dose exposure to PCP was demonstrated to be associated with abnormalities of the cellular and humoral immune parameters in humans [39]. A study conducted by Blakley et al. indicated that PCP suppressed the antibody response against sheep red blood cells by 39% when the response was expressed per viable spleen cell. This suppression was not evident when the response was expressed per spleen, suggesting that a compensatory mechanism or extramedullary splenic hemopoiesis was occurring, which minimized the overall impact on humoral immunity. The enhanced B- and T-lymphocyte blastogenesis may also reflect compensatory or hemopoietic activity [9]. In one of our previous studies, we demonstrated that both PCP and TCHQ were tumor promoters in spite of the different activity in inducing ROS, and exposure to PCP induced significant organ enlargement and lymphoma (spleen, liver and kidney) in mice [33]. Until now, the underlying mechanisms related to PCP-triggered lymphoma in mice remain unclear. Agents that are not mutagenic may induce direct toxicity with sustained tissue damage and subsequent cell proliferation. The cell proliferation resulting from toxicity may selectively induce enhanced replication of an already damaged genome in the initiated cell population. While cell toxicity does not directly induce carcinogenesis, it can enhance the process. Furthermore, a possible contribution in oxidative stress of PCP/TCHQ cannot be excluded from the promoting process via the activation of gene expression that leads to cell proliferation [90], [91]. Our current findings may partly explain the molecular mechanisms involved in the tumorigenesis of lymphoma in mice exposed to PCP. However, it should be noticed that PCP/TCHQ is able to damage the genome through oxidative attacks, leading to promutagenic lesions.

Taken together, the results of the present study demonstrated for the first time that TCHQ-induced necrosis of splenocytes may occur mainly through the massive ROS generation and also the ROS-triggered signaling pathway. The prolonged expression and activation of ERK may be involved in TCHQ-mediated necrosis. The effects may be mediated in part through the inhibition of apoptosis, whereas PCP primarily induced apoptosis rather than necrosis in splenocytes. To extent to which TCHQ induces apoptotic or nonapoptotic cell death (or both) is determined by complex factors, including the cell type and the environment surrounding the cells. Our results indicate that administration of the antioxidant NAC prevents ROS generation, cytotoxicity, and necrotic cell death in cells treated with TCHQ. Nonetheless, the detailed mechanisms of antioxidant in attenuating PCP/TCHQ-mediated immune toxicity are still unclear and need to be investigated further.
